# Optimized ancestral state reconstruction using Sankoff parsimony

**DOI:** 10.1186/1471-2105-10-51

**Published:** 2009-02-07

**Authors:** José C Clemente, Kazuho Ikeo, Gabriel Valiente, Takashi Gojobori

**Affiliations:** 1 Center for Information Biology and DNA Databank of Japan, National Institute of Genetics, Yata 1111, Mishima, Japan; 2 Technical University of Catalonia, E-08034 Barcelona, Spain

## Abstract

**Background:**

Parsimony methods are widely used in molecular evolution to estimate the most plausible phylogeny for a set of characters. Sankoff parsimony determines the minimum number of changes required in a given phylogeny when a cost is associated to transitions between character states. Although optimizations exist to reduce the computations in the number of taxa, the original algorithm takes time *O*(*n*^2^) in the number of states, making it impractical for large values of *n*.

**Results:**

In this study we introduce an optimization of Sankoff parsimony for the reconstruction of ancestral states when ultrametric or additive cost matrices are used. We analyzed its performance for randomly generated matrices, Jukes-Cantor and Kimura's two-parameter models of DNA evolution, and in the reconstruction of elongation factor-1*α *and ancestral metabolic states of a group of eukaryotes, showing that in all cases the execution time is significantly less than with the original implementation.

**Conclusion:**

The algorithms here presented provide a fast computation of Sankoff parsimony for a given phylogeny. Problems where the number of states is large, such as reconstruction of ancestral metabolism, are particularly adequate for this optimization. Since we are reducing the computations required to calculate the parsimony cost of a single tree, our method can be combined with optimizations in the number of taxa that aim at finding the most parsimonious tree.

## Background

Reconstruction of ancestral states aims at discovering the conformation of past proteins, genes or whole genomes from extant species data. This approach has been successfully utilized to reconstruct ancestral steroid receptors [1], mitochondrial DNA [2], antiviral RNase [3], or fluorescent proteins [4]. In a similar fashion, several studies have hypothesized on the evolution of hormone-receptor complexes [5], composition of ancestral genomes [6], thermostability of extinct proteins [7], properties of ancestral promoters [8], expansion of human segmental duplications [9], or ancestral codon usage [10].

Parsimony, maximum likelihood or bayesian approaches are commonly utilized to infer ancestral states. Parsimony was originally introduced by Edwards and Cavalli-Sforza [11], but its application to reconstruct ancestral characters was first described by Fitch [12]. Sankoff later proposed a modification to take into account different rates of change between states [13,14]. The popularity of likelihood methods in phylogenetics is mostly due to the optimization proposed by Felsenstein [15]. Yang described its application to infer ancestral sequences [16]. Bayesian approaches have also gained favor thanks to their combined used with Markov Chain Monte Carlo (MCMC) methods. Huelsenbeck and Bollback have proposed an algorithm for bayesian ancestral reconstruction [17].

Each of these approaches has advantages and weaknesses, and it is passionately debated which of them is more accurate. Parsimony is known for being biased when the rate of change per branch is high and tends to reconstruct the wrong tree due to long branch attraction [18], while likelihood does not suffer from these problems [19,20]. On the other hand, when the characters under study evolve at non-uniform rates over time, maximum likelihood and bayesian methods have been shown to be inconsistent and perform worse than parsimomy [21].

Regardless of the preferred method, the computational complexity of ancestral reconstruction algorithms is high and optimizations are required to work with large number of sequences. In the particular case of parsimony, an algorithm to reduce the the number of computations has been previously proposed [22]. Goloboff has introduced diverse optimization strategies [23,24], and Ronquist [25] has further improved some of the previous works. All these optimizations aim at reducing the calculations in the number of taxa when looking for the most parsimonious tree, that is, when looking for the tree in the search space that minimizes the number of changes. Nevertheless, no correct optimization in the number of states of the weighted parsimony algorithm proposed by Sankoff is known. Wheeler and Nixon proposed an optimization [26] later proved incorrect by Swofford and Siddall [27].

In this paper, we present a two-fold optimization of Sankoff parsimony. Our algorithm reduces the number of operations in the number of states needed to calculate the parsimony cost of a given phylogeny, as well as the time required to reconstruct the ancestral states. This optimization can be utilized when the cost matrix for transitions between character states is either ultrametric or additive, and it reduces the original *O*(*n*^2^) operations required with *n *states per node and character. While the optimization is moderate when the number of states is small, as in the case of nucleotides or amino acids, the optimization is more effective the larger the number of states, with an 8-fold reduction in running time in the case of metabolic enzymes. The algorithms here presented were originally developed precisely to obtain fast reconstructions of ancestral metabolism, motivated by the recent interest in obtaining phylogenetic signal from metabolic data [28-33].

In the rest of this paper we will review the original Sankoff algorithm, describe our optimization, and analyze its performance for both randomly generated data and biologically well-known cost matrices for nucleotides, amino acids, and metabolic enzymes.

## Methods

### Original Sankoff Parsimony

Sankoff parsimony [13,14] counts the number of evolutionary changes for a specific site in a phylogenetic tree, assuming a set of *n *character states *i *= 1, ..., *n *(for instance, 4 nucleotides or 20 amino acids) for which a cost matrix *C *= (*c*_*ij*_) of changes between states is given. Each node *p *of the phylogeny has assigned a cost vector, *S*^(*p*)^, which contains the minimal evolutionary cost $S_{i}^{\left(p\right)}$ for each of the character states. If node *p *is assigned state *i*, the quantity $S_{i}^{\left(p\right)}$ reflects the minimum cost of events (state changes) from *p *to the root of the tree.

The original Sankoff algorithm calculates the cost vectors at each node moving from the leaves upwards to the root. Initially, the *S*^(*x*) ^vectors at the inner nodes are unknown and those at the leaves are initialized with cost 0 for the observed character state and ∞ for the rest. For instance, if adenine is observed in a site (character) for a certain species, the cost vector would be $S_{a}^{\left(x\right)}$ = 0 and $S_{t}^{\left(x\right)}=S_{g}^{\left(x\right)}=S_{c}^{\left(x\right)}=\infty$. The cost vector of a node *p *with two children *q *and *r *is:

(1) $$S_{i}^{\left(p\right)}=\underset{j}{\min}(c_{ij}+S_{j}^{\left(q\right)})+\underset{k}{\min}(c_{ik}+S_{k}^{\left(r\right)})$$

Equation (1) states that the cost of being in character state *i *for node *p *is the cost of moving from character state *i *to *j *in child *q *(*c*_*ij*_) plus the cost of having reached state *j *at node *q *from the leaves ($S_{j}^{\left(q\right)}$). Character *j *is selected to minimize this sum, with the same procedure being applied to character *k *in child *r*. Algorithm 1 presents the original implementation of Sankoff parsimony to calculate the cost vector of all nodes in a tree for a single character.

**Algorithm 1 **(Original Sankoff algorithm: Up phase). *A procedure call Sankoff_Up*(*T*, *C*, *S*) *calculates the cost vector S*^(*p*) ^*of all nodes p of the phylogeny T, given a cost matrix C *= (*c*_*ij*_).

***procedure ****Sankoff_Up(T*, *C*, *S*)

   ***for all ****nodes p of T in postorder ****do***

      ***if ****p is a leaf ****then***

         ***for all ****i in *1, ..., *n ****do***

            ***if ****state i observed at leaf p ****then***

               $S_{i}^{\left(p\right)}$ ← 0

            ***else***

               $S_{i}^{\left(p\right)}$ ← ∞

      ***else***

         {*q*, *r*} ← *children of p*

         ***for all ****i in *1, ..., *n ****do***

            $S_{i}^{\left(p\right)}$ ← *cost*(*q*, *i*) + *cost*(*r*, *i*)

***function ****cost*(*x*, *i*)

   min ← ∞

   ***for all ****j in *1, ..., *n ****do***

      ***if ****c*_*ij *_+ $S_{j}^{\left(x\right)}$ < min ***then***

         min ← *c*_*ij *_+ $S_{j}^{\left(x\right)}$

   ***return ***min

**Figure 1:**
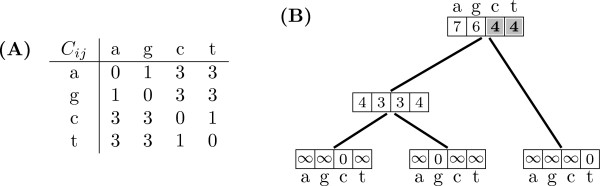
**Original Sankoff parsimony: up phase**. (A) A matrix defining transition costs between states. (B) A sample phylogenetic tree with cost vectors calculated for all nodes using Algorithm 1.

Figure 1 presents an example for a single site in a nucleotide sequence, where cytosine, guanine, and thymine are observed at the leaves. The minimum number of changes under Sankoff parsimony is 4, as annotated in the root of the tree. As it can be observed, Algorithm 1 takes *O*(*n*^2^) time in the number of character states per node, since the function *cost *is *O*(*n*) and it is called *n *times. Once the cost vectors have been calculated for each inner node, we can use this information to reconstruct the ancestral states. The algorithm proceeds now top-down, and the root of the phylogeny gets assigned those states with minimum cost in the vector *S*^(*root*)^. For any inner node *p*, and given that ancestral state *i *was reconstructed at its parent, the state *j *to be chosen is that for which *c*_*ij *_+ $S_{j}^{\left(p\right)}$ is minimized. Notice that for inner nodes this state needs not correspond with that for which $S_{j}^{\left(p\right)}$ is minimum. Algorithm 2 describes an implementation as presented in [[34], §6].

**Algorithm 2 **(Original Sankoff algorithm: Down phase). *A procedure call Sankoff_Down*(*x*, *T*, *C*, *S*, *S*_*anc*_) *calculates the ancestral states *$S_{anc}^{\left(p\right)}$*of all nodes p of the phylogeny T, given the root x of T, a cost matrix C *= (*c*_*ij*_) *of transition costs between states, and the cost vectors S*^(*p*) ^*for all nodes p of T as calculated by Sankoff_Up*(*T*, *C*, *S*).

***procedure ****Sankoff_Down(x, T, C, S, S*_*anc*_)

   $S_{anc}^{\left(x\right)}\leftarrow \arg\min_{i}S_{i}^{\left(x\right)}$

   ***for all ****j in *$S_{anc}^{\left(x\right)}$***do***

      ***for all ****child y of x ****do***

         *Sankoff_Down*(*j*, *y*, *T*, *C*, *S*, *S*_*anc*_)

***procedure ****Sankoff_Down*(*i*, *x*, *T*, *C*, *S*, *S*_*anc*_)

   *min_states*(*i*, *x*, *C*, *S*, *S*_*anc*_)

   ***for all ****j in *$S_{anc}^{\left(x\right)}$***do***

      ***for all ****child y of x ****do***

      *Sankoff_Down*(*j*, *y*, *T*, *C*, *S*, *S*_*anc*_)

***procedure ****min_states*(*i*, *x*, *C*, *S*, *S*_*anc*_)

   min ← ∞

   ***for all ****j in *1, ..., *n ****do***

      ***if ****x *= *root*(*T*) ***then***

         *trans_cost *← $S_{j}^{\left(x\right)}$

      ***else***

         *trans_cost *← *c*_*ij *_+ $S_{j}^{\left(x\right)}$

      ***if ****trans_cost *< min ***then***

         min ← *trans_cost*

         $S_{anc}^{\left(x\right)}$ ← {*j*}

      ***else if ****trans_cost *= min ***then***

         $S_{anc}^{\left(x\right)}$ ← $S_{anc}^{\left(x\right)}$ ∪ {*j*}

**Figure 2:**
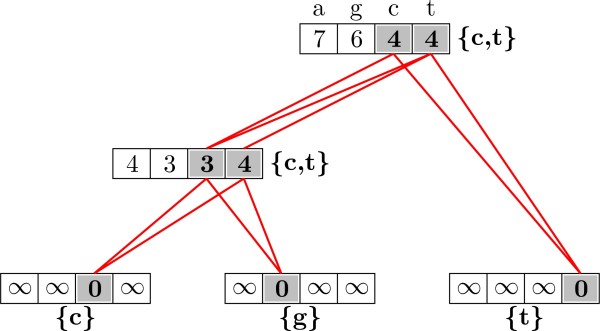
**Original Sankoff parsimony: down phase**. Ancestral states for the phylogeny, cost matrix and observed states of Figure 1, as obtained by Algorithm 2. Without solving ties, ancestral nodes are written next to each inner node. Red lines indicate the most parsimonious reconstructions after solving ties.

Figure 2 presents an example of the top-down phase of Sankoff parsimony. Notice that the states chosen as ancestral do not necesarily correspond to those with minimum values in the cost vectors. The node with cost vector *S *= (4, 3, 3, 4) has thymine as one of the two possible parsimonious ancestral states, which has larger cost than guanine. For simplicity, ties are not solved in the algorithms presented in this paper and thus inner nodes can have more than one ancestral state. If we were to differentiate among the different parsimonious reconstructions, there would be three scenarios for this example as indicated by the red lines: cytosine both at the root and the inner node, thymine both at the root and the inner node, or thymine at the root and cytosine at the inner node. Cytosine in the root and thymine in the inner node is not parsimonious. The reconstruction of ancestral states as described in Algorithm 2 takes *O*(*n*) time per node.

### Optimized Sankoff Parsimony

Our optimization is based on the observation that not all transition costs between character states need to be computed if the cost matrix is ultrametric or additive.

**Definition 1**. A cost matrix is ultrametric if for every three indices *i*, *j*, *k*, one of the three following inequalities holds (three point condition):

• *c*_*ij *_⩽ *c*_*ik *_= *c*_*jk*_

• *c*_*ik *_⩽ *c*_*ij *_= *c*_*jk*_

• *c*_*jk *_⩽ *c*_*ij *_= *c*_*ik*_

**Definition 2**. A cost matrix is additive if for every four indices *i*, *j*, *k*, ℓ, one of the three following inequalities holds (four point condition):

• *c*_*ij *_+ *c*_*k*ℓ _⩽ *c*_*ik *_+ *c*_*j*ℓ _= *c*_*i*ℓ _+ *c*_*jk*_

• *c*_*ik *_+ *c*_*j*ℓ _⩽ *c*_*ij *_+ *c*_*k*ℓ _= *c*_*i*ℓ _+ *c*_*jk*_

• *c*_*i*ℓ _+ *c*_*jk *_⩽ *c*_*ij *_+ *c*_*k*ℓ _= *c*_*ik *_+ *c*_*j*ℓ_

Notice that ultrametric matrices are also additive, since they satisfy the four point condition [35].

Consider a simple example where the cost matrix *C *has *c*_*ii *_= 0 and *c*_*ij *_= *k *for all *i *≠ *j*. When calculating the cost $S_{i}^{\left(p\right)}$ in Equation (1), we can substitute min_*j*_(*c*_*ij *_+ $S_{j}^{\left(q\right)}$) for min($S_{i}^{\left(q\right)}$, *k *+ min_*j*≠*i *_$S_{j}^{\left(q\right)}$), and similarly for the other child *r *of *p*. In the more general case of ultrametric or additive cost matrices, we can efficiently represent them with a unique rooted weighted cost tree *T*_*C *_using UPGMA [36] or neighbor-joining [37] respectively. The length of the path between any two leaves *i*, *j *in *T*_*C *_corresponds to the cost *c*_*ij*_. For ultrametric matrices, consider any set of leaves *L *= {*a*, *b*, ...} in the tree that have the same last common ancestor, *lca*(*L*). By definition, *lca*(*L*) is equidistant to any leaf in *L*, and all leaves in *L *are at the same distance *d *to each other (which is double the distance from the leaf to *lca*(*L*)). For any two leaves *a*, *b *in *L *we can then simplify the expression min_*L*_(*c*_*ab *_+ $S_{L}^{\left(q\right)}$) as *d *+ min $S_{L}^{\left(q\right)}$. Therefore, given the cost tree *T*_*C *_obtained by UPGMA from the ultrametric matrix, for each state *i *we only need to compute the minimum costs at the last common ancestor of that state and any other, that is, the inner nodes in the path from *i *to the root of *T*_*C*_. With additive matrices, since the distance from an inner node to its descendant leaves can vary, we need to take into consideration the specific length of each branch when calculating the minimum. Therefore, in our algorithm each cost vector *S*^*p *^is replaced by a cost tree $T_{C}^{\left(p\right)}$, whose inner nodes will contain the value that minimizes. *c*_*ij *_+ $S_{L}^{\left(q\right)}$ for all descendant leaves *L*. Algorithm 3 presents the optimized version of Sankoff parsimony for the calculations from the leaves to the root of the phylogeny.

**Algorithm 3 **(Optimized Sankoff algorithm: Up phase). *A procedure call Opt_Sankoff_Up*(*T*, *T*_*C*_, *S*) *calculates the cost vector S*^(*p*) ^*of all nodes p of the phylogeny T, given a cost tree T*_*C *_*representing an ultrametric or additive cost matrix C *= (*c*_*ij*_).

***procedure ****Opt_Sankoff_Up*(*T*, *T*_*C*_, *S*)

   ***for all ****nodes p of T in postorder ****do***

      ***if ****p is a leaf ****then***

         ***for all ****i in *1, ..., *n ****do***

            ***if ****state i observed at leaf p ****then***

               $S_{i}^{\left(p\right)}$ ← 0

            ***else***

               $S_{i}^{\left(p\right)}$ ← ∞

            *update*(*p*, *i*, $S_{i}^{\left(p\right)}$, *T*_*C*_)

      ***else***

         {*q*, *r*} ← *children of p*

         ***for all ****i in *1, ..., *n ****do***

            $S_{i}^{\left(p\right)}$ ← *cost*(*q*, *i*) + *cost*(*r*, *i*)

            *update*(*p*, *i*, $S_{i}^{\left(p\right)}$, *T*_*C*_)

***procedure ****update*(*x*, *i*, *v*, *T*_*C*_)

   *n *← *leaf of *$T_{C}^{\left(x\right)}$*corresponding to state i*

   *cost*(*n*) ← *v*

   *min_tags*(*n*) ← {*i*}

   *cost *← *branch length between n and its parent in *$T_{C}^{\left(x\right)}$

   ***repeat***

      *n *← *parent of n in *$T_{C}^{\left(x\right)}$

      ***if ****v *+ *cost *<*cost*(*n*) ***then***

         *cost*(*n*) ← *v *+ *cost*

         *min_tags*(*n*) ← {*i*}

      ***else if ****v *+ *cost *= *cost*(*n*) ***then***

         *min_tags*(*n*) ← *min_tags*(*n*) ∪ {*i*}

      ***if ****n *≠ *root of *$T_{C}^{\left(x\right)}$***then***

         *cost *← *cost *+ *branch length between n and its parent in *$T_{C}^{\left(x\right)}$

   ***until ****n *= *root of *$T_{C}^{\left(x\right)}$

***function ****cost*(*x*, *i*)

   *n *← *leaf of *$T_{C}^{\left(x\right)}$*corresponding to state i*

   min ← *cost*(*n*)

   *cost *← *branch length between n and its parent in *$T_{C}^{\left(x\right)}$

   ***repeat***

      *n *← *parent of n in *$T_{C}^{\left(x\right)}$

      ***if ****cost *+ *cost*(*n*) < min ***then***

         min ← *cost *+ *cost*(*n*)

      ***if ****n *≠ *root of *$T_{C}^{\left(x\right)}$***then***

         *cost *← *cost *+ *branch length between n and its parent in *$T_{C}^{\left(x\right)}$

   ***until ****n *= *root of *$T_{C}^{\left(x\right)}$

   ***return ***min

Algorithm 3 utilizes a cost tree $T_{C}^{\left(p\right)}$ with the same topology as *T*_*C *_for each node *p*, and where each node is annotated with the minimum value corresponding to *c*_*ij *_+ $S_{L}^{\left(q\right)}$, as implemented in the function *cost*. The function *update *saves each of these cost trees by moving from the leaves to the root and storing minimum values in the nodes (*cost*(*n*)), as well as the leaf responsible for the value stored in the node (*min_tags*(*n*)), which will be later used to optimize the reconstruction of ancestral states. The complexity of Algorithm 3 depends on the internal path length of *T*_*C*_. The worst case would be a degenerate tree with linear structure, in which case the complexity for *n *states is (*n*^2 ^- *n*)/2 per node and character [[38], §2.3.4.5]. Notice that in practice this will be a rare case, and most cost trees have a more favourable topology to our optimization (as will be shown in the Results section), while the original algorithm takes *O*(*n*^2^) time no matter the cost matrix used.

**Figure 3:**
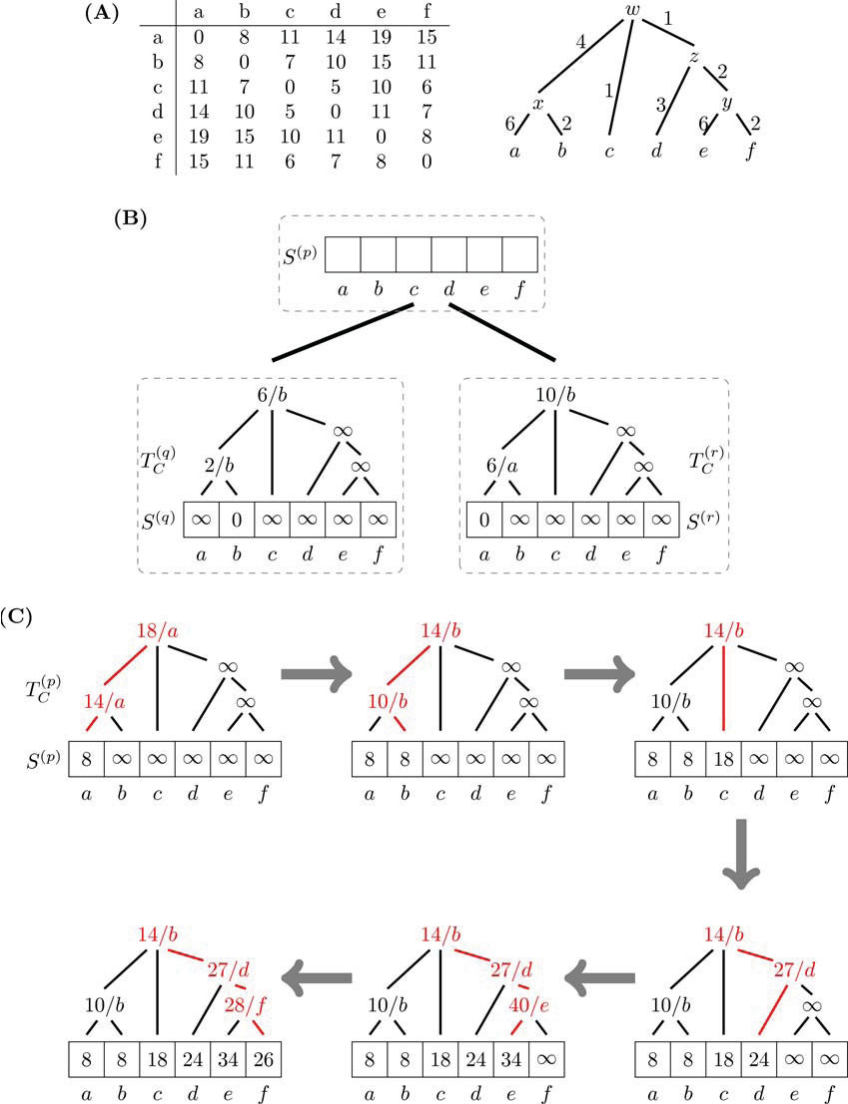
**Optimized Sankoff parsimony: up phase**. (A) Cost matrix between six states *a*, ..., *f *and their associated cost tree as calculated by the neighbor-joining algorithm. (B) A simple phylogeny with three nodes (dashed boxes), their cost vectors *S *and cost trees *T*_*C*_. Cost vectors *S*^(*q*) ^and *S*^(*r*)^, and cost trees $T_{C}^{\left(q\right)}$ and $T_{C}^{\left(r\right)}$ are already calculated. (C) Step-by-step reconstruction of *S*^(*p*) ^and $T_{C}^{\left(q\right)}$ for node *p *following Algorithm 3.

Figure 3 presents a detailed example of the Algorithm 3. The cost of a transition between states *i *and *j*, *c*_*ij*_, is the sum of branch lengths between the corresponding leaves in the cost tree. Since the matrix in this example is additive, neighbor-joining guarantees a unique tree. The minimum cost for state *a *can be calculated as $S_{a}^{\left(p\right)}$ = *cost*(*q*, *a*) + *cost*(*r*, *a*), the sum of minimum costs from the children nodes *q *and *r*. For node *q *this cost will be the minimum among ∞ (cost at leaf *a *in $T_{C}^{\left(q\right)}$), 8 (cost 2 at node *x *plus branch length 6), and 16 (cost 6 at node *w *plus total branch length 6 + 4), therefore *cost*(*q*, *a*) = 8. For node *r*, clearly *cost*(*r*, *a*) = 0, and therefore $S_{a}^{\left(p\right)}$ = 8. At this point, we update the path from leaf *a *to the root of $T_{C}^{\left(p\right)}$: node *x *is set to 8 + 6 = 14, and node *w *is set to 8 + 6 + 4 = 18. The cost for state *b *is calculated following the same procedure, resulting in $S_{b}^{\left(p\right)}$ = 8. When updating $T_{C}^{\left(p\right)}$, the cost at node *x *is reduced from 14 to 10 (8 at leaf *b *plus branch length 2), and at node *w *from 18 to 14. The remaining state costs and inner nodes of the cost tree are calculated in a similar way.

Algorithm 4 presents the optimized version of the reconstruction of ancestral states using Sankoff parsimony. In its original implementation, for each node we had to consider all posible states looking for the one that minimized *c*_*ij *_+ $S_{j}^{\left(x\right)}$. Since we have already saved the minimum transition costs and the leaves responsible for them as shown in Algorithm 3, we can use this information to further speed up computation. The ancestral states for the root *r *of the phylogeny *T *are obtained as in the original algorithm. For any inner node *x *of *T*, and given that its parent had ancestral state *k *reconstructed, we only need to move from leaf *k *to the root of $T_{C}^{\left(x\right)}$ and keep the state *min_tags*(*n*) that has the minimum value *cost*(*n*).

**Algorithm 4 **(Optimized Sankoff algorithm: Down phase). *A procedure call Opt_Sankoff_Down*(*x*, *T*, *T*_*C*_, *S*_*anc*_) *calculates the ancestral states *$S_{anc}^{\left(p\right)}$*of all nodes p of the phylogeny T, given the root x of T and the cost tree *$T_{C}^{\left(p\right)}$*for each node p of T as calculated by Opt_Sankoff_Up*(*T*, *T*_*C*_, *S*).

***procedure ****Sankoff_Down*(*x*, *T*, *T*_*C*_, *S*_*anc*_)

   $S_{anc}^{\left(x\right)}\leftarrow \arg\min_{i}S_{i}^{\left(x\right)}$

   ***for all ****j in *$S_{anc}^{\left(x\right)}$***do***

      ***for all ****child y of x ****do***

         *Opt_Sankoff_Down*(*j*, *y*, *T*, *T*_*C*_, *S*_*anc*_)

***procedure ****Opt_Sankoff_Down*(*i*, *x*, *T*, *T*_*C*_, *S*_*anc*_)

   *n *← *leaf of *$T_{C}^{\left(x\right)}$*corresponding to state i*

   *min *← *cost*(*n*)

   *cost *← *branch length between n and its parent in *$T_{C}^{\left(x\right)}$

   ***repeat***

      *n *← *parent of n in *$T_{C}^{\left(x\right)}$

      ***if ****cost *+ *cost*(*n*) < min ***then***

         min ← *cost *+ *cost*(*n*)

         $S_{anc}^{\left(x\right)}$ ← {*min_tags*(*n*)}

      ***else if ****cost *+ *cost*(*n*) = min ***then***

         $S_{anc}^{\left(x\right)}$ ← $S_{anc}^{\left(x\right)}$ ∪ {*min_tags*(*n*)}

      ***if ****n *≠ *root of *$T_{C}^{\left(x\right)}$***then***

         *cost *← *cost *+ *branch length between n and its parent in *$T_{C}^{\left(x\right)}$

   ***until ****n *= *root of *$T_{C}^{\left(x\right)}$

   ***for all ****j in S*_*anc*_***do***

      ***for all ****child y of x ****do***

         *Opt_Sankoff_Down*(*j*, *y*, *T*, *T*_*C*_, *S*_*anc*_)

**Figure 4:**
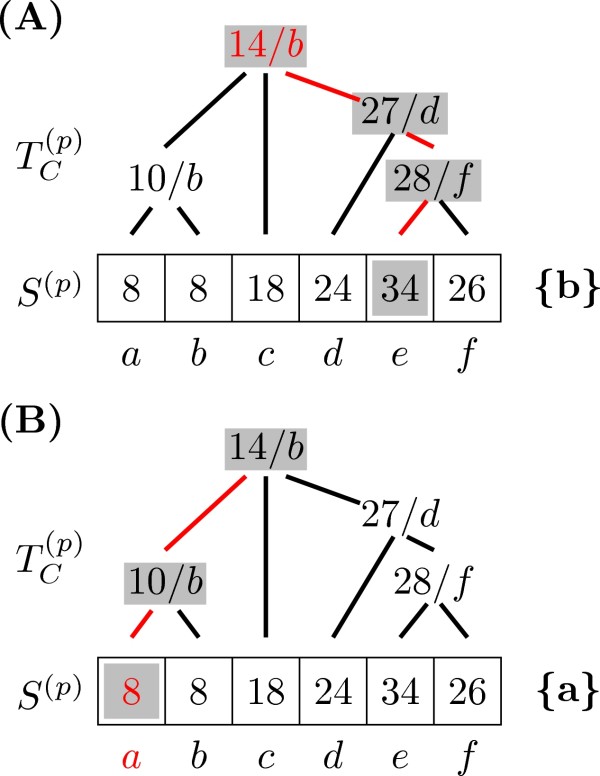
**Optimized Sankoff parsimony: down phase**. (A) Calculation of the ancestral state for node *p *following Algorithm 4, given that the parent node of *p *had ancestral state *e*. (B) Calculation of the ancestral state for node *p *following Algorithm 4, given that the parent node of *p *had ancestral state *a*.

Figure 4 presents an example of Algorithm 4. Assuming the parent node of *p *had state ancestral state *e*, we move from that leaf to the root of $T_{C}^{\left(p\right)}$ while comparing minimum values: 34 at *e*, 34 (28 + 6) if we move from state *f*, 35 (27 + 6 + 2) if we move from *d *or 25 (14 + 6 + 2 + 1) when moving from *b*. The ancestral state reconstructed in this node is therefore *b*. If the ancestral state at the parent had been *a *instead, node *p *would have state *a *as the most parsimonious. Notice that in this case the value in the root of $T_{C}^{\left(p\right)}$ is not necessary, since the cost of moving from *b *to *a *is in fact 10 (minimum annotated at the parent of *a*) plus 6 (the length of the branch from *a *to its parent).

Algorithm 4 reduces the number of operations compared to the original implementation, since we do not have to review all states at each node but only traverse from a leaf to the root of the cost tree. The original implementation takes *O*(*n*) time, while the complexity of our optimization is again a function of the internal path length of *T*_*C*_, which in general will be less than *n*.

## Results and Discussion

**Figure 5:**
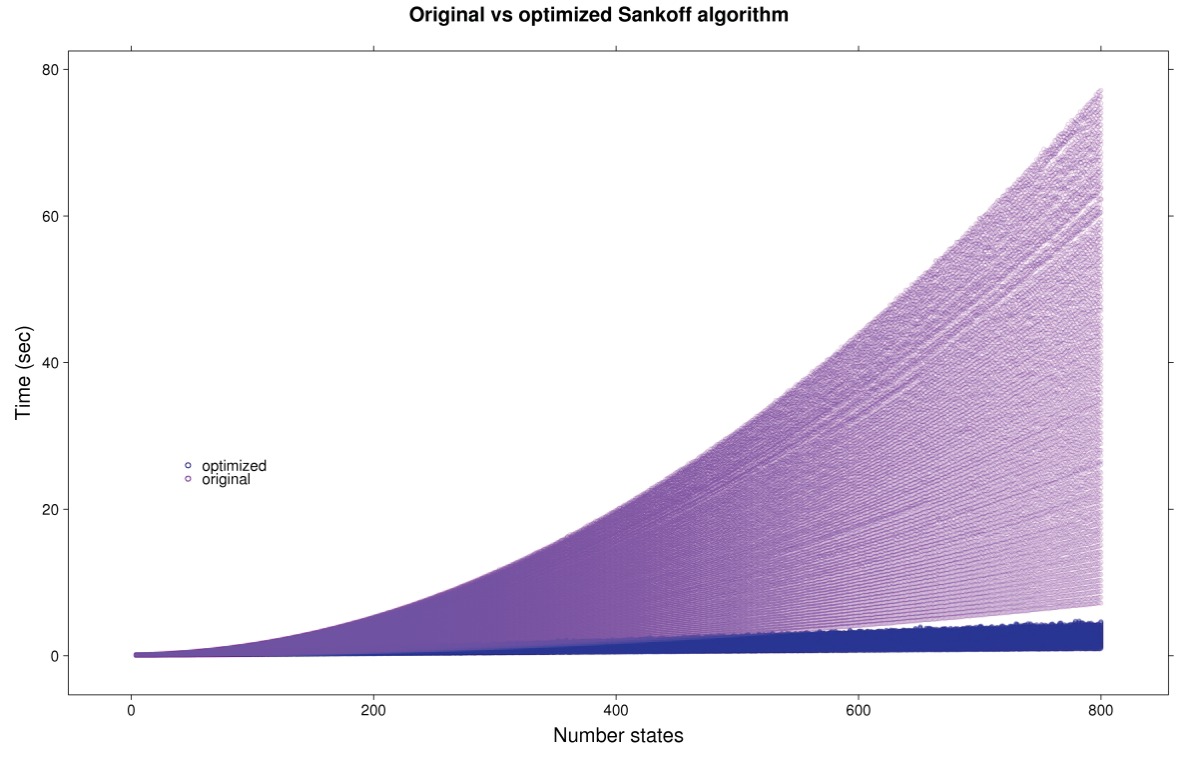
**Execution times for simulated cost matrices**. Execution time versus number of states (4 to 800) with random cost matrices and phylogenies (10 to 100 species), with the original and optimized Sankoff parsimony algorithms.

We performed a series of simulations involving a single random site from 10 to 100 species in a random phylogeny, and 4 to 800 character states. Sankoff parsimony costs were calculated using randomly generated additive matrices and their associated cost trees. All experiments were performed in a Mac Pro with 2 × 2.66 Ghz Dual-Core Intel Xeon processor, 7 GB of memory, and Mac OS X 10.4.11. Figure 5 presents running times versus the number of states, with the running time shown for all the generated phylogenies; that is, for each point corresponding to a particular number of states in the horizontal axis, there are 91 measurementes (10 to 100 species) of execution times for both the original and optimized methods. As it can be observed, times for the optimized algorithm grow linearly in the number of states, while the original implementation has a quadratic growth. Results for ultrametric matrices (not shown) were similar to those presented in Figure 5.

**Figure 6:**
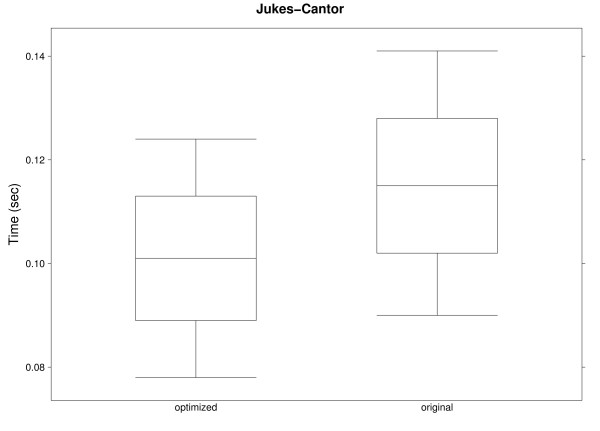
**Execution times for Jukes-Cantor model**. Execution times for one nucleotide site in 91 phylogenies (10 to 100 species) with the original and optimized implementations of Sankoff parsimony, using a cost matrix based on the Jukes-Cantor model.

**Figure 7:**
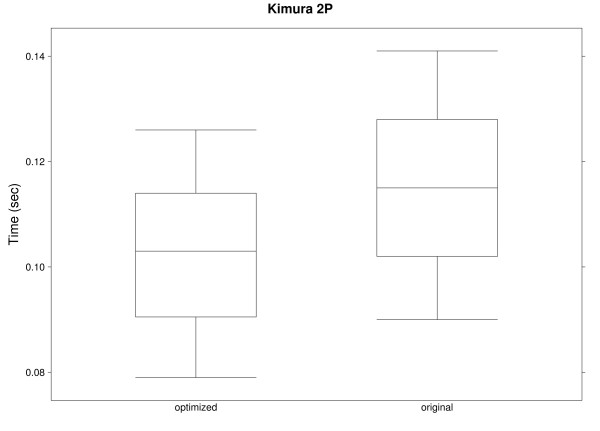
**Execution times for Kimura two-parameter model**. Execution times for one nucleotide site in 91 phylogenies (10 to 100 species) with the original and optimized implementations of Sankoff parsimony, using a cost matrix based on Kimura's two-parameter model.

Figures 6 and 7 show results for a single randomly generated nucleotide and phylogeny (10 to 100 species) using cost matrices based on the DNA evolution models proposed by Jukes and Cantor [39] and Kimura [40], both of which are ultrametric. Even though there are only 4 character states, the optimized algorithm outperforms the original implementation. Notice that results for these two figures and the previous one are for a single site; calculations for full sequences would further increase the difference between the original and the optimized algorithms.

**Figure 8:**
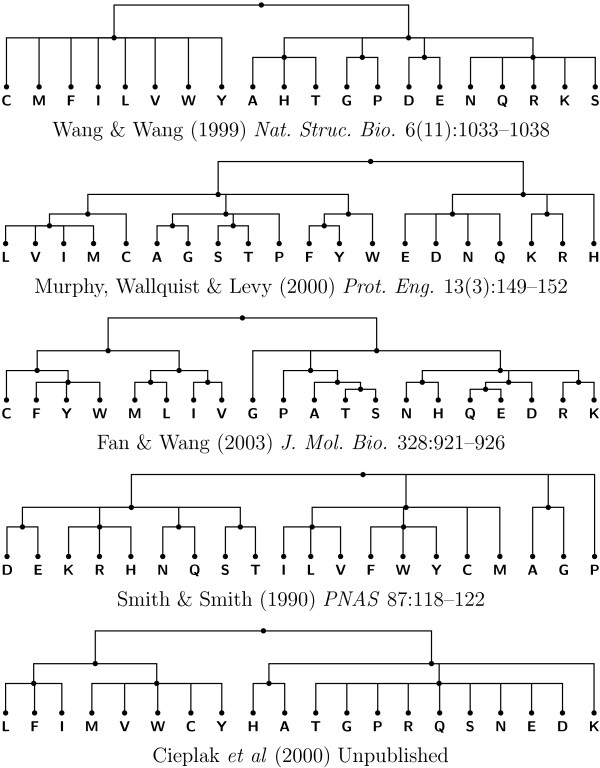
**Amino acid cost trees**. Cost trees corresponding to five different amino acid cost matrices [42-46].

**Figure 9:**
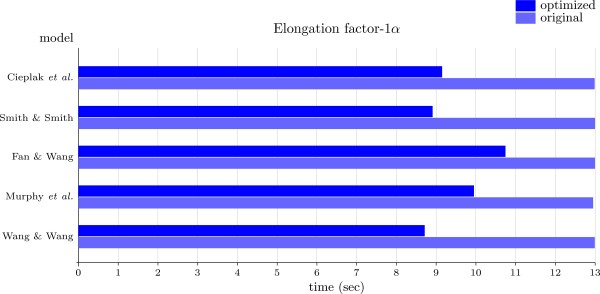
**Execution times for ancestral elongation factor-1*α *reconstruction**. Execution times for the reconstruction of elongation factor-1*α *(EF-1*α*) in 42 species, with the cost trees presented in Figure 8. EF-1*α *sequences obtained from [41].

To provide a better idea of the performance of our optimization in a more realistic setup, we reconstructed the ancestral amino acid chain of elongation factor-1*α *from the sequence of 42 species (see [41] for details). The cost matrices were obtained from works addressing the problem of how to obtain a reduced amino acid alphabet that can still produce a correct folding [42-46]. Figure 8 presents the corresponding cost trees. As it can be seen in Figure 9, in this particular example the optimization is approximately 27% faster than the original algorithm.

**Figure 10:**
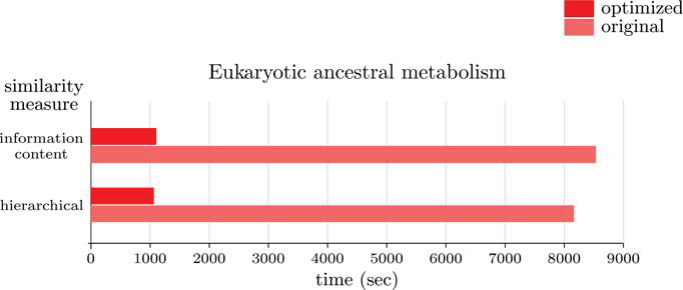
**Execution times for ancestral eukaryotic metabolic reconstruction**. Execution times for the reconstruction of ancestral metabolism in a group of twelve eukaryotes, those described in [47] plus *Xenopus laevis*, *Candida albicans*, *Cyanidioschyzon merolae*, *Danio rerio*, and *Oryza sativa japonica*.

Figure 10 presents results for the reconstruction of the ancestral metabolism of twelve eukaryotes (adapted from [47], with *Xenopus laevis*, *Candida albicans*, *Cyanidioschyzon merolae*, *Danio rerio*, and *Oryza sativa japonica *added). Hierarchical and information content similarity measures [48] were used to determine the transition costs between states (in this case, enzymes identified by their EC number [49]). The characters to reconstruct will be the enzymatic reactions annotated to the species in KEGG [50], with enzymes representing the possible states. Whenever an enzymatic reaction is not annotated to a species, its state will be chosen as the subset of annotated enzymes that are closer to it, at the corresponding cost. This is in order to avoid having cost vectors in the leaves with all their entries having cost ∞. For instance, under hierarchical similarity, if the enzyme alcohol dehydrogenase (1.1.1.1) is not annotated to a species, we would look first for any annotated enzyme in the group of oxidoreductases acting on the CH-OH group of donors with *NAD*^+ ^or *NADP*^+ ^as acceptor (1.1.1.-), which are the closest to 1.1.1.1 at cost 0.25. If no such enzyme is annotated, we would look for those in the group 1.1.-.- (cost 0.5 to 1.1.1.1), and so on until a group with annotated enzymes for the species is found.

Alternatively, we could have codified the presence or absence of enzymes in each species, and then perform a reconstruction using maximum parsimony. While this is a commonly used approach, the use of measures of enzymatic similarity has shown a better performance than simple patterns of presence/absence of enzymes in the phylogenetic analysis of metabolism [31], and therefore the election of large state sets is to be preferred. For this particular example, the number of states is composed of 925 reactions annotated to at least one of the twelve species under study, and the number of inner nodes in the cost tree is comparatively small, making our optimization 8-fold faster than the original implementation.

## Conclusion

The optimization here presented provides a computation of Sankoff parsimony faster than the original algorithm when the cost matrix is ultrametric or additive, even for a small number of character states. Since our approach reduces the execution time needed to calculate the parsimony cost of a single tree, it could be easily combined with optimizations looking for the most parsimonous tree. Our algorithm takes comparatively less time to execute when the number of states is large, and therefore problems such as ancestral metabolism reconstruction could be especially well-suited for this optimization.

## Authors' contributions

JC and GV conceived the method and prepared the manuscript. JC implemented the algorithms and performed the experiments. All authors contributed to the discussion and have approved the final manuscript.
